# A precision cryostat design for manual and semi-automated cryo-plunge instruments

**DOI:** 10.1063/1.4967864

**Published:** 2016-11

**Authors:** Christopher J. Russo, Steve Scotcher, Martin Kyte

**Affiliations:** MRC Laboratory of Molecular Biology, Francis Crick Avenue, Cambridge CB2 0QH, United Kingdom

## Abstract

Here we describe a bench-top cryostat system to control the temperature of liquid ethane in a cryo-plunge apparatus designed for biological specimen preparation for electron cryomi-croscopy. It comprises a foam insulated Dewar containing a copper cryostat cup, whose temperature is controlled via an active feedback system to within 0.1 K. The device can easily be incorporated into existing manual and semi-automatic cryo-plunge instruments that are not equipped with cryogenic temperature control. Over the course of normal use, we find that using a cryostat is convenient, fast, and does not require special mixtures of cryogens like ethane/propane. This simple cryostat improves the reliability and reproducibility of biological specimen preparation for electron cryomicroscopy.

## I Introduction

Electron cryomicroscopy (cryo-EM) depends on the ability to freeze water quickly enough to ensure it enters an amorphous solid phase.^1^ The recent advances in the field have vastly increased the number of specimen being prepared for high-resolution structural analysis and the number of laboratories using cryo-preparation methods to vitrify specimens for imaging with electrons.^2^,^3^ This is usually achieved using the method developed by Dubochet and colleagues, by rapidly plunging a thin (typically less than one micrometer thick, limited by the thermal conductivity of water) biological specimen into a liquid cryogen near its melting point, at a temperatures near that of liquid nitrogen at its boiling point (77.4 K).^1^,^4^,^5^ In the intervening years, several different instruments have been developed to perform this rapid freezing process, and each incorporates varying degrees of automation and offers some control over the parameters of the process like plunge speed, humidity surrounding the specimen, blot time, and force.^1^,^6^–^11^ Other more complicated techniques like freeze-fracture deep etching, high-pressure freezing, and slam freezing are also used for the preparation of specimen for electron microscopy^12^,^13^ but require more complicated instruments and are beyond the present scope.

The most commonly used cryogen for freezing is liquid ethane, for the following reasons: 1. the melting point (90.4 K) is slightly above the boiling point of liquid nitrogen, 2. the heat capacity (68.5 J/mol K) is similar to water (74.5 J/mol K), and 3. the boiling point is 184 K, giving a large range of temperature before the liquid loses thermal contact with a surface by boiling, thus avoiding calefaction and the Leidenfrost effect.^5^,^11^ Ethane is inexpensive and readily available, which has led to its wide adoption as a cryogen used for specimen preparation in electron cryomicroscopy.

Still, ethane has several drawbacks, including its flammability, its potential to cause burns if it contacts exposed tissue, and its melting point being above that of liquid nitrogen. While the first two hazards are easily mitigated by careful handling and safe protocols, the latter leads to reduced reproducibility and reliability in the specimen preparation process. It is desirable to keep the ethane temperature close to its melting point as this ensures proper vitrification of the specimen and prevents the formation of crystalline ice.^1^ This presents a practical problem during cryo-preparation since the ethane will freeze solid if it comes into contact with liquid nitrogen or is indirectly cooled to near liquid nitrogen temperatures with a container which is in thermal contact with liquid nitrogen. To avoid this problem, users of plunge-freeze instruments typically need to provide, periodically, some source of heat to raise the temperature of the ethane to above 90.4 K. Often this involves inserting a metal implement, like a copper rod or pair of tweezers, into the frozen ethane to melt it and raise the temperature.^11^ If the temperature is raised too much, then the specimen will not be cooled fast enough to vitrify or will crystallise after vitrification, rendering it unsuitable for imaging. This process requires some time and skill and can therefore reduce the speed and reliability with which specimens are vitrified on the supports. Further, it also creates an opportunity for cross-contamination between specimen via the implements used to heat the ethane.

One way to avoid some of these drawbacks is to use cryogens other than ethane, but these too have limitations. Propane (melting point 85.5 K, boiling point 231 K, and specific heat capacity 73.6 J/mol K) can also be used but has the disadvantage that in its solid form, it sublimes in vacuum at a lower rate than ethane and this may affect the amount of residual material left on the specimen.^1^ Chlorodifluoromethane (Freon-22) is a inert, non-flammable cryogen with a melting point of 97.7 K and boiling point of 232.5 K.^5^ Historically, it was used along with other chlorofluorocarbons for freezing specimen,^6^ but due to their detrimental environmental impact, the use of Freons has been phased out in most countries.^14^ Another option is to use specified mixtures of ethane and propane.^15^ By adding ethane to propane, the melting point is lowered; at a mixture of about 37%:63% ethane:propane, the melting point drops below liquid nitrogen and thus will remain liquid even at 77.4 K. But obtaining special gas mixtures is sometimes difficult and often involves long lead times. In a laboratory that does a large amount of cryo-specimen preparation, this can be a limiting factor. And there still is some uncertainty in when the cryogen has reached temperature as this can vary depending on the Dewar configuration and condition.

To avoid these problems while still allowing the use of a cheap and relatively innocuous cryogen like ethane, some commercial freeze-plunge apparatus like the Leica EM GP and the EMS-002 have built-in, proprietary temperature controllers for their ethane cups with varying degrees of temperature stability. Still, there are a vast number of custom made manual plungers and semi-automated instruments without temperature control which would benefit from having a robust cryostat that eliminates temperature as a variable in the specimen freezing process. This includes the original plungers designed by Dubochet and colleagues at European Molecular Biology Laboratory (EMBL),^1^ the manual plunger design of Talmon and colleagues,^7^ which has been in continuous use at the MRC-LMB for 25 yr, other home-built plungers constructed for specific types of experiments at various laboratories,^8^,^9^,^16^,^17^ and the popular FEI Vitrobot which was originally designed by Frederik and colleagues^10^ and is now developed and sold by FEI, Inc. With this in mind, we sought to make a simple ethane cryostat system that can be retrofitted to any manual or semi-automated plunge system. While the design is specifically made for the FEI Vitrobot and the custom-built manual plungers of Talmon type, the principles of the design can be easily adapted to other freezing instruments and cryostats in general.

## II Instrument Design

**Fig. 1:**
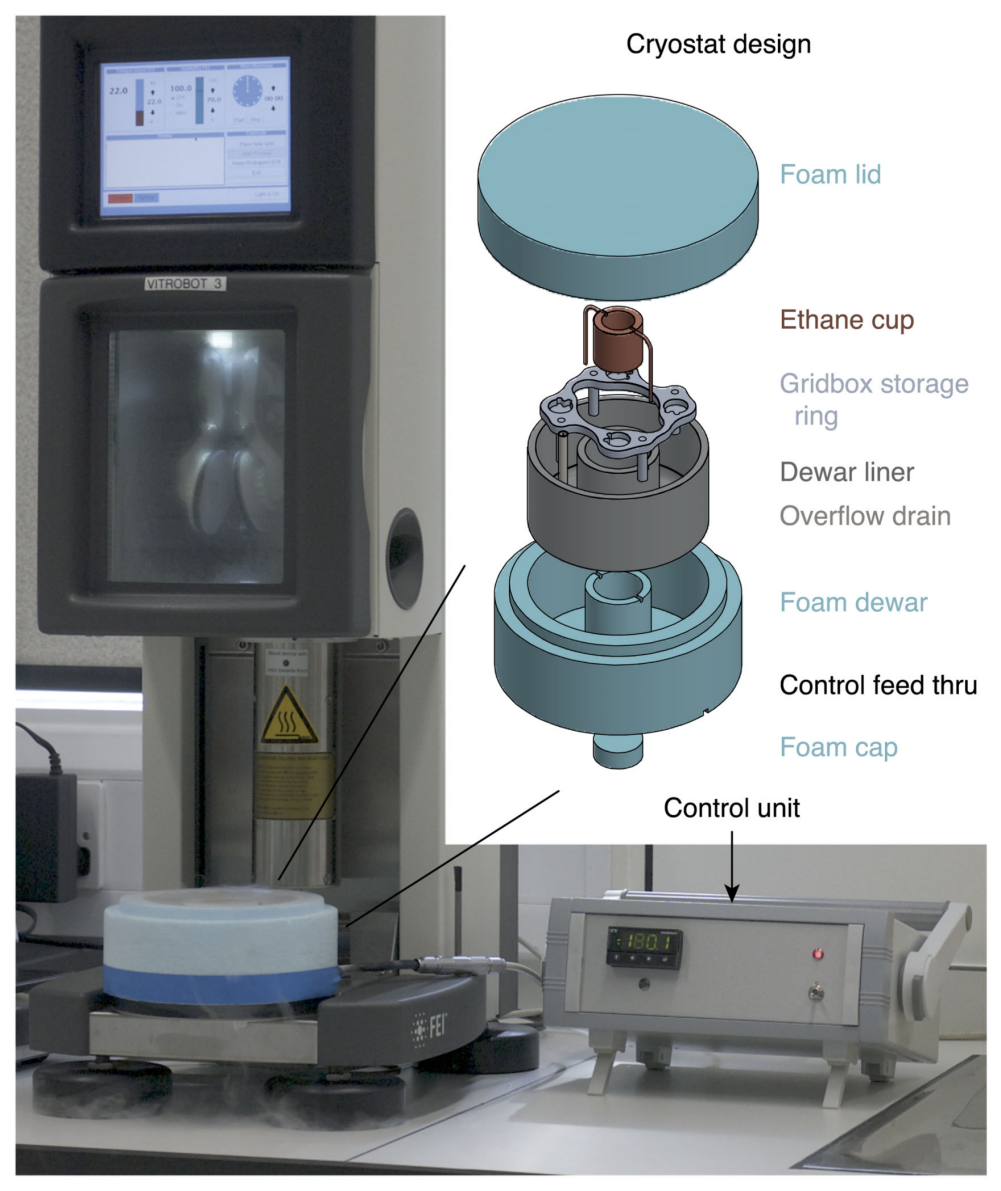
Cryostat system. Photo of the cryostat system on a Vitrobot Mk IV (FEI) semi-automated plunge-freeze instrument attached with a cable to the electronic control unit. Inset shows an exploded view diagram of the components of the Dewar.

The overall design of the cryostat system is shown in Fig. 1. An insulating Dewar containing a reservoir of liquid nitrogen surrounds an inner insulating ring, which contains a copper cup to hold the liquid ethane used to freeze the specimen. The temperature of the ethane cup is controlled by balancing the cooling power of two copper cooling arms submersed in liquid nitrogen, with heating power provided by two 10 W cartridge heaters imbedded in the walls of the cup. The outer ring of the Dewar provides a storage point for grid boxes and a reservoir of liquid nitrogen for cooling both the ethane cup and the stored grids. An overflow spout is fitted within the outer ring to control the liquid nitrogen level which prevents overfilling.

**Fig. 2:**
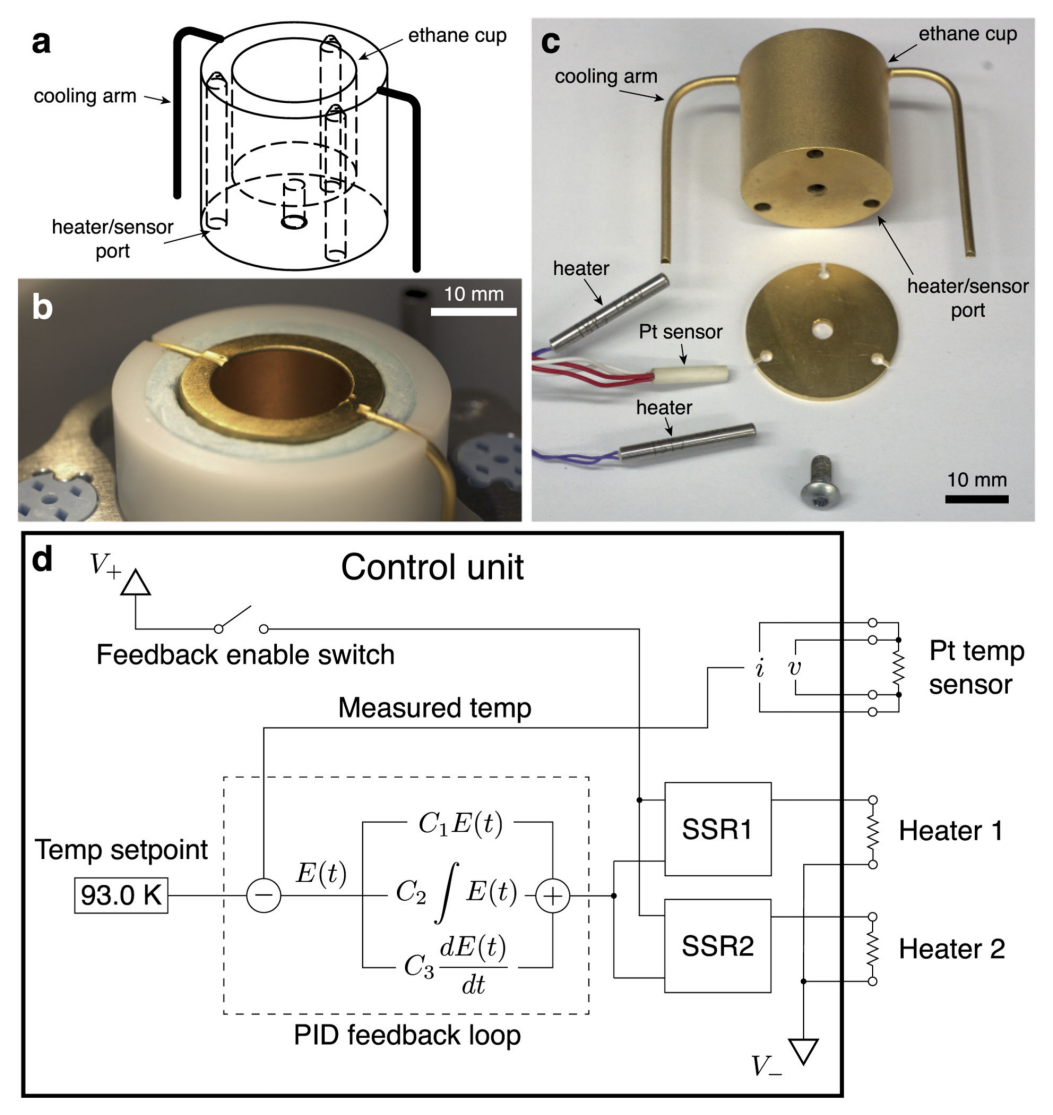
Cryogen temperature control system. The design of the gold plated copper ethane cup (a) and its implementation ((b) and (c)) are shown. The copper cup is insulated from the outer container of liquid nitrogen (b) such that during use, the heat is removed through the two cooling arms. Heating power is provided by two resistive cartridge heaters inserted into the copper cup from below (c). The temperature sensor is located away from both the heaters and the cooling arms to ensure uniform heating of the cup. The cooling arms are silver soldered to the monolithic copper cup and have a specific cross section to control the heat transfer, determined empirically, as described in the text. A diagram of the feedback system in the electronic control unit is shown in (d).

To measure the temperature of the ethane cup, a calibrated platinum resistor is located within the sidewall of the cup and is attached to the control electronics using a 4-wire probe configuration to improve the accuracy of the measurement (Fig. 2). The heating elements are positioned away from the sensor and each other to ensure uniform temperature across the ethane cup. The cup is made of copper—chosen because of its high thermal conductivity—and is electroplated with a layer of nickel (2 *μ*m thick adhesion layer) and a final layer of gold (0.5 *μ*m thick surface layer), to prevent oxidation, reduce the emissivity of the surface, and ensure the durability and easy cleaning of the cup. The sensor and heater connections are attached with a shielded cable to a small control box, which is used to both set and monitor the temperature of the cryostat.

The overall design of the control unit is diagrammed in Fig. 2(d). It comprises a DC power supply, used to drive the 10 W cartridge heaters, and a proportional-integral-differential (PID) feedback control loop, attached to a digital display interface, which is used to display and set the temperature. The PID loop is tuned to minimise the settling time using the feedback constants (*C*_1_, *C*_2_, *C*_3_ in Fig. 2(d)) in an automated routine. This feedback loop drives two solid state relays (SSRs), which control the current delivered to the heating elements.

The most important factor in achieving the level of temperature stability demonstrated below was the design of the cooling arms (Figs. 2(a)-2(c)). Ideally, the balance between the amount of cooling and the amount of heating of the ethane cup should be such that at the desired set-point, the duty cycle or amount of heating power is half its maximum value. This results in maximal response performance and a wide range of stable temperature around the set-point. To achieve this, we first estimated the cooling power of a copper rod of the appropriate length (from the cup to the mid-level of nitrogen in the outer ring) and then estimated the total cross sectional area required to give 10 W of cooling. Since other factors like the thermal conduction through the solder joints can also affect the cooling power, we then tested the design using a prototype cup with copper braids to provide the cooling. The braids were larger in cross section than required; during the testing we carefully cut away the strands of the braid until we reached the desired 50% power for ethane at its melting point. We then measured the cross sectional area of the remaining braid and used this to set the final dimensions of the copper cooling arms (Fig. 2(c)).

## III Results and Discussion

**Fig. 3:**
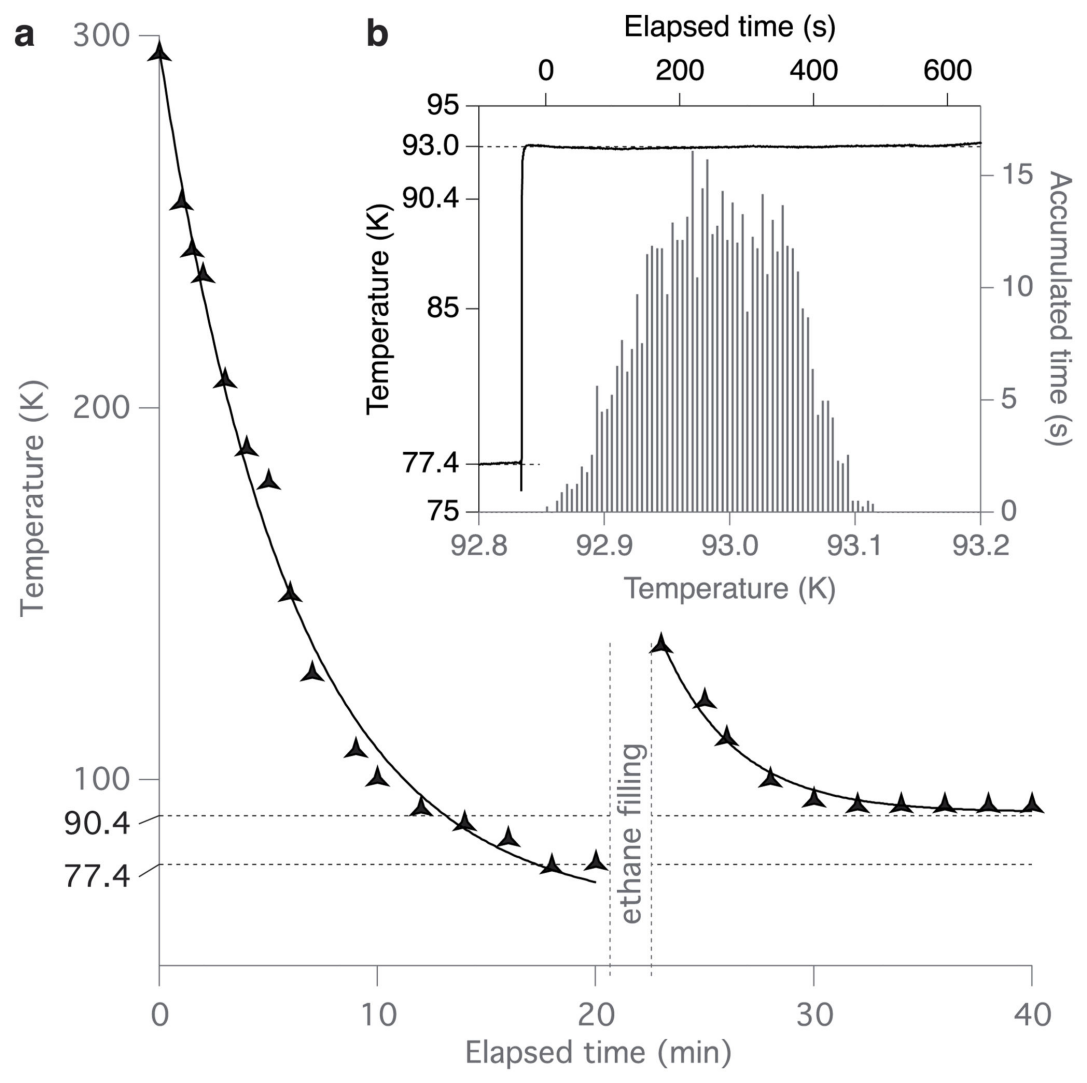
System performance. Plot of cryostat temperature during cooling from room temperature (a). Solid lines are exponential fits to the data. The system goes below the melting point of ethane (90.4 K) in about ten minutes and reaches the control temperature set point (93.0 K) after filling the ethane cup in less than ten minutes. Inset plot (b) shows the temperature of an external probe placed in the ethane (step in the plot) during a continuous recording where the set temperature was 93.0 K. Histogram shows the temperatures of the probe during a 10 min period under feedback control with no user input to the system (from 0 to 600 in the time trace). Over this period, the mean temperature of the ethane was 92.99 ± 0.05 (standard deviation).

Cooling the system is achieved by filling the outer container ring with liquid nitrogen, covering it with the lid, and waiting for the ethane to reach the desired temperature. This process can be sped up by repeatedly topping up the ethane cup and outer ring with nitrogen during cooling. A standard procedure for the use of the instrument is provided in the supplementary material. The cooling speed of the cryostat under typical use was measured and is plotted in Fig. 3(a). The ethane cup cools to <100 K in about ten minutes, following an exponential trajectory with time constant 6.0 ± 0.4 min. After cooling the feedback loop is enabled, and the ethane cup is filled by liquefying ethane gas, which is slowly delivered into the cup from a cylinder through a regulator and nozzle. The entire setup takes 20-30 min, mostly unattended, and requires 0.5 l of liquid nitrogen and 7 ml (4 g) of liquid ethane. This means that a single type-J gas cylinder (20 × 71 cm) containing 13 kg of ethane can provide more than 300 fills or approximately one year’s worth of cryogen for daily use. During active use, the liquid nitrogen consumption rate is about 0.25 l/h and significantly less with the heating turned off. Fitting the lid and giving occasional top-ups allow one to use the cryostat for an entire day without warming if desired, as the snugly fitting lid will inhibit frost formation within the instrument when closed.

To test the temperature stability, the cryostat was cooled as described, filled with ethane, and the feedback controller was set to 93.0 K, which is the typical operating temperature for freezing grids. A calibrated K-type thermocouple probe was taken from liquid nitrogen at 77.4 K and placed in the centre of the liquid ethane. The temperature was recorded for 10 min and is shown in Fig. 3(b). Over the course of a ten minute test (0-600 s in the time trace), the mean temperature was 92.99 ± 0.05 (standard deviation). A histogram of the temperature during this interval is shown in Fig. 3(b). This demonstrates that both the accuracy and precision of the temperature control are less than 0.1 K. Even though the precision of the temperature control would allow one to keep the temperature even closer to the ethane melting point (90.4 K), in practice keeping it a couple degrees above prevents solidification upon splashing with liquid nitrogen when refilling the outer ring. During active use, the reservoir should be topped up approximately every ten minutes for optimal temperature stability.

**Fig. 4:**
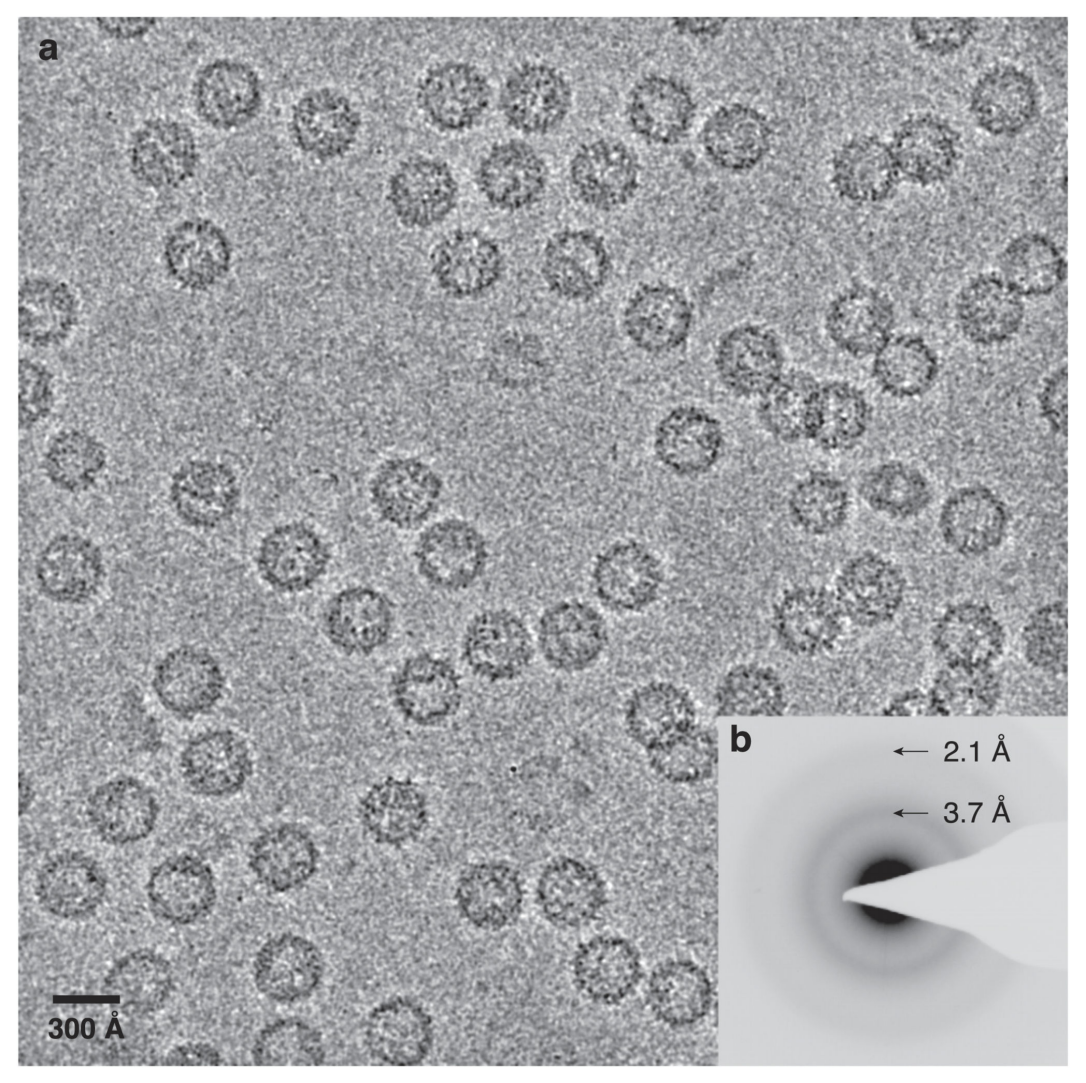
Example specimen. Micrograph (a) of a vitrified preparation of purified hepatitis B viral capsids, frozen using the precision cryostat at 93.0 K. Imaged with 300 keV electrons using a fluence of 50 e^−^/Å^2^ during a 2.0 s exposure at 83 K on a Falcon 3 direct electron detector in integrating mode. A selected area (≃500 nm in diameter, 470 mm camera length) electron diffraction pattern (b) of the same specimen show in (a) shows amorphous rings with maxima at (3.7 Å)^−1^ and (2.1 Å)^−1^, which are the defining property of vitreous ice.^1^

To ensure that the system is effective and practical for routine use, we froze numerous types of specimen (>10), including pure water and aqueous solutions of protein complexes, DNA, RNA, and viruses on multiple types of specimen supports^18^ (all-gold, bare copper, Quantifoil Cu/amC, and 700 mesh gold grids without a support foil) over several months and have had no difficulty in reproducibly preparing thin films of amorphous ice suitable for high-resolution imaging. An example is shown in Fig. 4, where a specimen of purified hepatitis B capsids was vitrified using a standard, previously published protocol^19^ with a Vitrobot Mk III, an UltrAufoil specimen support^20^ and reagent grade ethane (BOC 99% pure, N2.0 grade). The cryostat was operated according to the protocol described in the supplementary material, with Vitrobot plunge instrument settings as specified in the previously published protocol.^19^ The ice was uniform across the grid, and showed no evidence of surface or embedded contaminants. To demonstrate that the ice is in the amorphous phase, we collected a selected area electron diffraction pattern of the frozen specimen suspended within the holes of the support (Fig. 4(b)). The ring pattern of the diffracted electrons and the lack of any reflection spots indicate that the water ice is in the amorphous state.^1^ The cryostat has been tested by several other users with varying amounts of experience preparing specimen—from a few months to a few decades—and no significant faults were found with the system design in day-to-day use.

Finally, we note that occasionally, a thin layer of opaque material collects on the surface of the liquid ethane during use. These are contaminants (not solid ethane, since the temperature is accurately controlled, thus preventing any solid ethane formation) which can end up on the surface of the frozen specimen and interfere with imaging. The precise control of the ethane temperature, afforded by this device, allows one to detect the presence of these contaminants and remove them. Without temperature control, the solid contaminants can easily be confused with frozen ethane, making them difficult to detect and eliminate.

## IV Conclusions

Precisely controlling the freezing temperature eliminates one variable from the cryo-EM specimen preparation process, thus reducing the difficulty and improving the reproducibility of making high-quality specimens for high-resolution imaging. Further, it obviates the need for mixtures of ethane and propane or repeated manual heating of the cryogen during cryo-EM specimen vitrification. The cryostat design delineated herein uses simple yet robust principles of feedback control and can be adapted to the design of cryostats in other applications.

## Supplementary Material

See the supplementary material for a detailed protocol for using the cryostat for the preparation of vitreous specimen for electron microscopy.

Supp Info
