# Supplementary material for: A precision cryostat design for manual and semi-automated cryo-plunge instruments
Source: Rev Sci Instrum. Author manuscript; Available in PMC 2017 Jan 27. (PMC5270774; doi:10.1063/1.4967864)
Supplement: Supp Info [file NIHMS71125-supplement-Supp_Info.pdf]

Supplementary material
MRC-LMB Precision cryostat procedure

Reference: A precision cryostat design for manual and semi-automated cryo-plunge instruments, Russo *et al.* 2016.

Christopher J. Russo
MRC Laboratory of Molecular Biology
Cambridge CB2 0QH UK
`crusso@mrc-lmb.cam.ac.uk`

Summary

This procedure describes how to use the LMB ethane cryostat for the FEI Vitrobots or manual cryo-plunge instruments. The cryostat comprises a foam insulated, liquid nitrogen Dewar with a central cryogen plunge cup with temperature control, and an electronic control box used to display and set the desired temperature. The design allows the temperature of the plunge container to be controlled from 77K to 114K with a precision of 0.1K.

Warning: This procedure involves hazardous and flammable chemicals at cryogenic temperatures. It is for use by experienced scientists who know how to handle such chemicals and instruments and have done all appropriate local safety training and risk assessments, etc. Use at your own risk, and always wear safety glasses!

Detailed procedure

1. Before beginning, remove the lid and clean the specimen box tray (aluminium) and the inner Dewar with ethanol and a clean, lint free wipe (e.g. Tek-Wipe or lens paper, not Kimwipe). Let dry completely in air.
2. Attach the control cable to the Dewar (red dot aligns with red dot on connector) and *then* turn on the controller (switch on back of box).
3. From here on MAKE SURE YOU WEAR SAFETY GLASSES! One drop of liquid ethane in your eye can cause blindness. Do not take this lightly.
4. Fill the outer ring and the cryogen cup with clean, ice-free liquid nitrogen. Wait for it to boil off, then fill again and apply the lid. You can then monitor the temperature on the controller. It takes about 3 Dewar volumes of nitrogen (~0.5 L) and a few minutes to cool it to $<$100K. Once the temp is below 100 K (-173 °C) and the cup is dry (no liquid nitrogen) you can begin filling with ethane.
5. Turn on the temperature control switch on the right of the front panel. If the display says OPER press the right button until it says RUN.
6. For ethane, the recommended set temperature is 93.0K (-180.1 °C) which is just slightly above the melting point (90.4K), and there is no need to ever change this unless you are using a different cryogen. Turn on the temperature control before filling to ensure the ethane in the tip will not freeze and clog during filling.
7. Purge the fill line briefly and then begin filling the cup with ethane. Start very slowly, and then you can increase the flow with the valve when a puddle of liquid ethane has formed.
8. Fill the cup to just below the top, so the ethane still has a slight negative meniscus.
9. Top up the nitrogen and then cover with the lid. As the ethane cools down to 93K it will shrink and perhaps wick up the surface of the metal so you may need to top it up slightly once it reaches temperature.
10. Once at the set point temperature, the tuned PID feedback loop will keep the temperature constant to within 0.1K. The diode under the temperature display lights up when the heat is on.
11. Apply the lid until you are ready to use. This will prevent frost formation in the Dewar. The hold time is one hour before the outer ring must be topped up.
12. During use, be sure to keep the grids in the thin layer of vapour during transfer from the cup to the storage boxes to prevent de-vitrification and contamination. The flat side should be towards the rear of the Vitrobot. The outer ring should be topped up every 10 minutes during active use for optimum stability.
13. Occasionally, a thin layer of opaque material collects on the surface of the liquid ethane. These are contaminants (not solid ethane, since the temperature control prevents solid ethane formation) which can end up on the surface of the specimen. The simplest way to remove them is by gently skimming the surface of the ethane with a piece of filter paper (Whatman No. 1). If the problem persists or is particularly troublesome one should check the cleanliness of the containers and the source of ethane for possible sources of contamination.
14. When finished, turn off the temperature control (front), turn off the power (rear) and then unplug the Dewar, in that order.
15. Let the Dewar warm up in a fume hood, again wipe down the surfaces with ethanol and then store warm and dry with the lid on.
